# Thermal expression of intersubjectivity offers new possibilities to human–machine and technologically mediated interactions

**DOI:** 10.3389/fpsyg.2014.00802

**Published:** 2014-07-23

**Authors:** Arcangelo Merla

**Affiliations:** Infrared Imaging Lab, Department of Neuroscience, Imaging and Clinical Sciences–Institute of Advanced Biomedical Technologies, G. d’Annunzio UniversityChieti–Pescara, Italy

**Keywords:** human–machine interaction, psychophysiology, thermal infrared imaging, emotions, intersubjectivity

## Abstract

The evaluation of the psychophysiological state of the interlocutor is an important element of interpersonal relationships and communication. Thermal infrared (IR) imaging has proved to be a reliable tool for non-invasive and contact-less evaluation of vital signs, psychophysiological responses, and emotional states. This technique is quickly spreading in many fields, from psychometrics to social and developmental psychology; and from the touch-less monitoring of vital signs and stress, up to the human–machine interaction. In particular, thermal IR imaging promises to be of use for gathering information about affective states in social situations. This paper presents the state of the art of thermal IR imaging in psychophysiology and in the assessment of affective states. The goal is to provide insights about its potentialities and limits for its use in human–artificial agent interaction in order to contribute to a major issue in the field: the perception by an artificial agent of human psychophysiological and affective states.

## INTRODUCTION

We routinely interact with machines since they pervade our lives. Over the centuries, the way we interact has dramatically changed since the machines have evolved from pure mechanical tools to complex robots endowed with humanoid capabilities. If we refer to machine as every non-human non-biological actor able to passively or actively interact with humans, the fields of human–machine interaction (HMI), human–computer interaction (HCI), and human–robot interaction can be unified into the general field of human–artificial agents interaction (HAI).

A common key challenge of all typologies of the artificial agents (AA) is to set up a contingent interaction. This means that AA not only must react to human actions, but also that they must (or should) react in ways that are congruent with the emotional and psychophysiological state of the human user or interlocutor. The latter aspect is especially relevant for social and affective robots, which are designed to interact with human users in a variety of social context and over long periods of time. Such AA need to communicate with people in ways that must be promptly comprehended and accepted (Kirby et al., 2010).

Affective state, mood, and emotion play an important role in social interaction. Emotional responses are triggered by social interactions, influenced by cultural and societal patterns, and expended to communicate desires to other people (Parkinson, 1996). Emotions bring colloquial content, consenting conversational partners to increase the effectiveness of their communication (Clark and Brennan, 1991). For example, the desire or the need to be comforted may be expressed through a manifestation of sadness that may be facial, vocal, or behavioral. Moreover, the actual mood of a person may have an effect on the way that person interacts with others (Forgas, 1999). People who are interacting may unconsciously tune moods and emotions to match those of their conversational partner (Wild et al., 2001). Cover-up of emotions can be highly disadvantageous for forming relationships and is disruptive to conversations (Butler et al., 2003). In fact, the principal reason for social interaction is to experience emotions, which help to develop a “sense of coherence with others” (Frijda, 2005).

People tend to treat AA as they treat other people, attempting to establish a social relationship with them (Reeves and Nass, 1996). Therefore, the above-mentioned “sense of coherence with others” defines the core of need for congruency of the HAI. Understanding the psychophysiological state of other individuals plays an essential role for planning or adopting congruent strategies in social interactions. Such an innate capability is at the basis of empathetic sharing among humans. To give AA this capability is one of the most important challenges in the field of the HAI (Pantic and Rothkrantz, 2003). However, recognition and instrumental measuring of affective states is also one of the most challenging research activities in the field of applied psychophysiology.

## ASSESSMENT OF PSYCHOPHYSIOLOGICAL STATES THROUGH THERMAL INFRARED IMAGING

To date, monitoring of psychophysiological and emotional states is usually performed through the measurements of several autonomic nervous system (ANS) parameters, like skin conductance response, hand palm temperature, heartbeat, and/or breath rate modulations, and peripheral vascular tone. This assessment is also performed through behavioral channels, like facial expression recognition and electromyography activity. Classical technology for monitoring autonomic activity usually requires contact sensors or devices, resulting somehow invasive and potentially biasing the estimation of the state, as the compliant participation of the individual is required.

Thermal infrared (IR) imaging was proposed as a potential solution for non-invasive and ecological recording of ANS activity (Merla et al., 2004). Thermal IR imaging, in fact, allows the contact-less and non-invasive recording of the cutaneous temperature through the measurement of the spontaneous thermal irradiation of the body.

The autonomic nervous system is fundamentally involved in the bioheat exchange, unconsciously controlling heart rate, breathing, tissue metabolism, perspiration, respiration, and cutaneous blood perfusion. It provides an effective tool for observations of emotional responses and states. Previous research in this field has demonstrated that thermal IR imaging (also referred to as functional infrared imaging, fIRI) can characterize competing subdivisions of the ANS (Murthy and Pavlidis, 2006; Garbey et al., 2007; Merla and Romani, 2007; Pavlidis et al., 2007; Shastri et al., 2009; Merla, 2013; Engert et al., 2014). Since the face is usually exposed to social communication and interaction, thermal imaging for psychophysiology is performed on the subject’s face. Given the proper choice of IR imaging systems, optics, and solutions for tracking the regions of interest, it is possible to avoid any behavioral restriction of the subject (Dowdall et al., 2006; Zhou et al., 2009).

The reliability and validity of this method was proven by comparing data simultaneous recorded by thermal imaging and by golden standard methods, as ECG, piezoelectric thorax stripe for breathing monitoring or nasal thermistors, skin conductance or galvanic skin response (GSR). As for the latter, studies have demonstrated that fIRI and GSR have a similar detection power (Coli et al., 2007; Shastri et al., 2009; Pavlidis et al., 2012; Di Giacinto et al., 2014; Engert et al., 2014).

An almost exclusive feature of thermal IR imaging in stress research is its non-invasiveness. In a recent study, Engert et al. (2014) explored the reliability of thermal IR imaging in the classical setting of human stress research. Thermal imprints were compared to established stress markers (heart rate, heart rate variability, finger temperature, α-amylase, and cortisol) in healthy subjects participating into two standard and well-established laboratory stress tests: the cold pressor test (Hines and Brown, 1932) and the trier social stress test (Kirschbaum et al., 1993). The thermal responses of several regions of the face proved to be change sensitive in both tests. Although the thermal imprints and established stress marker outcome correlated weakly, the thermal responses correlated with stress-induced mood changes. On the contrary, the established stress markers did not correlate with stress-induced mood changes. These results suggest that thermal IR imaging provides an effective technique for the estimation of sympathetic activity in the field of stress research.

The maturity and the feasibility achieved by thermal IR imaging suggest its use even in psychiatry or psychophysiology (Merla, 2013). Recently, thermal IR imaging was used, together with standard GSR, to examine fear conditioning in posttraumatic stress disorder (PTSD; Di Giacinto et al., 2014). The authors examined fear processing in PTSD patients with mild symptoms and in individuals who did not develop symptoms (both groups consisting of victims of a bank robbery), through the study of fear-conditioned response. The authors found: (a) a change of physiological parameters with respect to the baseline condition in both control subjects and PTSD patients during the conditioning phase; (b) the permanence of the conditioning effect in the maintenance phase in both control and PTSD patients; and (c) patients and controls did differ for the variation across the phases of the physiological parameters rather than for their absolute values, showing that PTSD patients had a prolonged excitation and higher tonic component of autonomic activity. These results indicate that the analysis of facial thermal response during the conditioning paradigm is a promising psychometric method of investigation, even in the case of low level of PTSD symptom severity.

Thermal IR imaging was indicated as a potential tool to create, given the use of proper classification algorithms, an atlas of the thermal expression of emotional states (Khan and Ward, 2009; Nhan and Chau, 2010). This would be based on the characterization of the thermal signal in facial regions of autonomic valence (nose or nose tip, perioral or maxillary areas, periorbital, and supraorbital areas associated with the activity of the periocular and corrugator muscle, and forehead), to monitor the modulation of the autonomic activity.

The above-mentioned studies were possible, thanks to the impressive advancement of the technology for thermal IR imaging. Modern devices ensure a high spatial resolution (up to 1280 × 1024 pixels with up to a few milliradiants in the field-of-view), high temporal resolution (full-frame frequency rate up to 150 Hz), and high thermal sensitivity (up to 15 mK at 30°C) in the spectral range [3÷5] μm (Ring and Ammer, 2012). The commercial availability of 640 × 480 focal plane array of uncooled and stabilized sensors (spectral range 7.5÷13.0 μm; full-frame frequency rate around 30 Hz; thermal sensitivity around 40 mK at 30°C) permits the extensive use of this technology in the psychophysiological arena.

However, several limitations exist for using thermal IR imaging in a real world and everyday life scenario. Because of the homeostasis, the cutaneous temperature is continuously adjusted to take into account the environmental conditions. Cautions and countermeasures must therefore be adopted to avoid attributing any psychological valence to pure thermoregulatory or acclimatization processes (Merla et al., 2004).

## THERMAL EXPRESSIONS OF INTERSUBJECTIVITY

According to Kappas (2013), “emotions are evolved systems of intra- and interpersonal processes that are regulatory in nature, dealing mostly with issues of personal or social concern.” Emotions regulate social interaction and the social sphere. According to Kappas (2013), social processes impact and regulate emotions. This means that “intrapersonal processes project in the interpersonal space, and inversely, interpersonal experiences deeply influence intrapersonal processes.” These reciprocal connections between interpersonal and intrapersonal emotions and processes are important elements for achieving interaction awareness.

**FIGURE 1:**
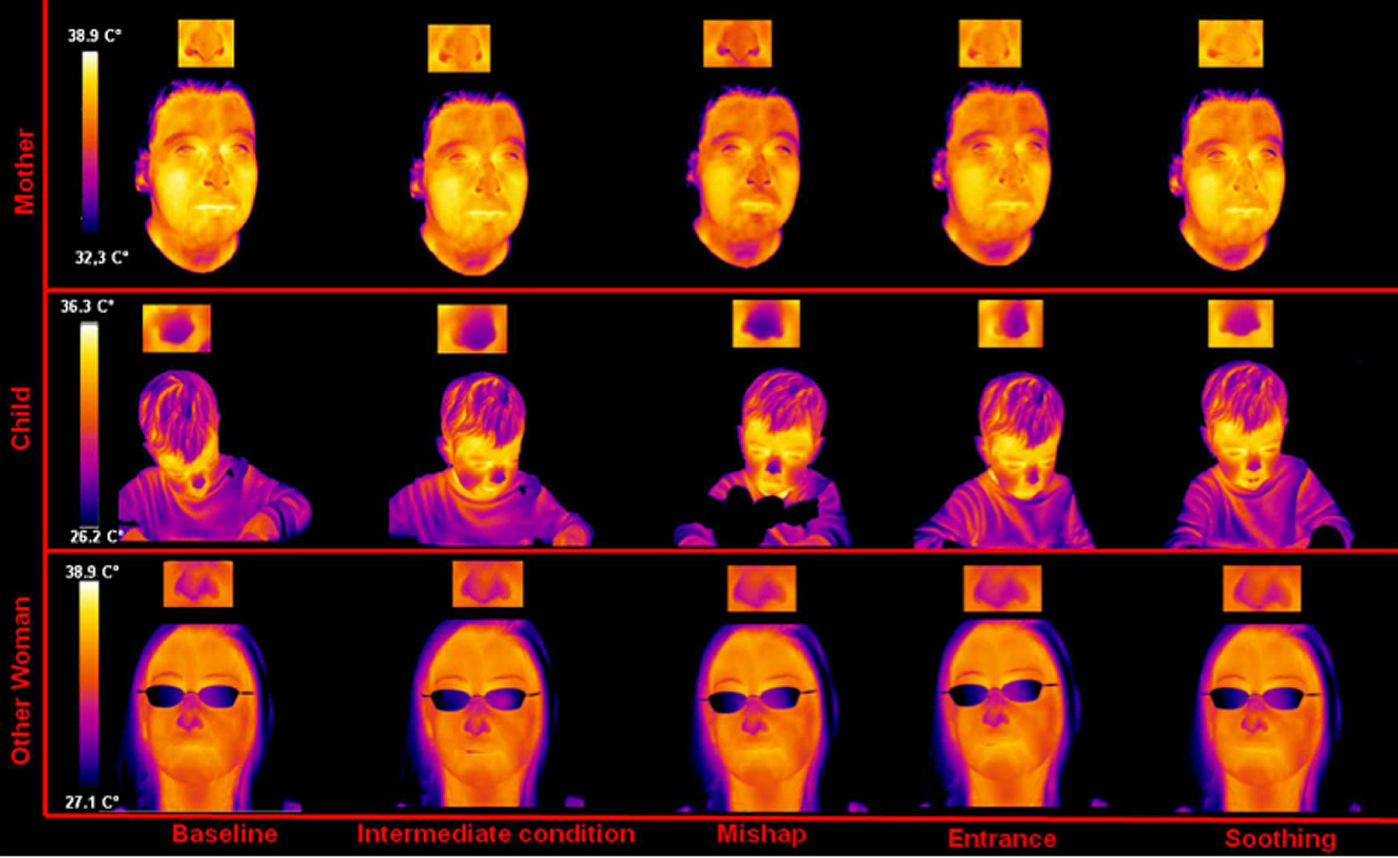
**Facial thermal imprints of a mother–child–other mother triad synchronization during distressing situation (adapted from Manini et al., 2013).** The picture shows many of the features of thermal imaging in psychophysiology, especially the possibility of simultaneous recording several individuals sharing an experimental condition or a social interaction. Facial temperature variations are shown across experimental phases. Such variations, expression of the sympathetic activity may regard not only the average value but also the spatial distribution of the temperature across the regions of interest.

However, as outlined above, emotions may posses a thermal signature or may be characterized by a regulatory activity of the autonomic nervous system, which in turn possesses a thermal imprint through which it can be detected. In addition, the thermal modulation of real and natural social interaction among individuals can be studied non-invasively through thermal IR imaging, even recording thermal signatures from more individuals at once (**Figure 1**). Therefore, it is plausible to talk in terms of thermal expression of emotions and interaction as a channel for studying intersubjectivity intended as psychological relation between people. Studies in this field have regarded mostly maternal empathy and social interaction (Ebisch et al., 2012; Manini et al., 2013).

Early infant attachment was studied using thermal IR imaging in infants exposed to three different experimental phases: (i) separation from the mother; (ii) a short-lived replacement of the mother by a stranger; and (iii) infant in the presence of the mother and the stranger. By observing temperature changes on the infants’ forehead, the researchers concluded that infants are aware of strangers and that infants form a parental attachment earlier than previously thought, specifically from 2 to 4 months after birth (Mizukami et al., 1990).

Maternal empathy is considered fundamental to develop affective bonds and a healthy socio-emotional development. Ebisch et al. (2012) demonstrated that a situation-specific parallelism between mothers’ and children’s facial temperature variations exists (**Figure 1**). This study was the first that proved evidence, in a pure natural context, for a direct affective sharing involving autonomic responding.

An extension of the above study with an additional group of female participants showed that mothers–child dyads in contrast to other-women–child dyads have faster empathic reactions to the child’s emotional state (Manini et al., 2013). As for the adults, fewer studies of social interaction with thermal IR imaging are available.

Merla and Romani (2007) exposed the participants to the attention of unknown people, while performing a stressful task (a stroop test). The study was designed in order to elicit feeling of embarrassment and mild stress when the participants failed to perform correctly the task in the presence of others. Temperature decreases associated with emotional sweating were observed on the palm and the face, especially around the mouth and over the nose tip. The authors reported that the largest temperature variations were found for those subjects more influenced by the presence of unknown people, while less significant variations were found in subjects less interested in the judgment of others.

Given the capability of thermal IR imaging to capture emotional states, a variety of studies examined the potential of this technique in the context of deception detection (Pavlidis et al., 2002; Tsiamyrtzis et al., 2006; Zhou et al., 2009). Often, individuals who commit a crime show involuntary physiological responses when remembering details of that crime. By capitalizing on the thermal imprint of such responses, Pollina et al. (2006) found significant facial temperature differences between deceptive and non-deceptive participants.

Sexual arousal has clear and marked interrelationships with ANS activity. Merla and Romani (2007) studied the facial thermal response of healthy males to the view of erotic video clips in contrast with the view of sport movies. Through bioheat models, these facial temperature variations were converted into cutaneous perfusion variations and compared with the penis response, measured through a pneumatic device. Cutaneous perfusion of specific facial regions (nose, lips, and forehead) markedly increased during sexual-based content video more than during non-sexual-based stimuli.

Hahn et al. (2012) examined social contact and sexual arousal during interpersonal physical contact. This study investigated facial temperature changes with interpersonal social contact. The stimulus was a standardized interaction with a same-and opposite-sex experimenter touching the subject over face and chest (high-intimate contact) and arm and palm (low-intimate contact). Facial temperatures significantly increased from baseline during the high-intimate contact, these temperature increases being larger when an opposite-sex experimenter touched the subject. The study demonstrated that facial temperature changes were reliable indicators of arousal during interpersonal interactions.

## THERMAL IR IMAGING AND ARTIFICIAL AGENT PERCEPTION

In recent years, the robotics community has increased the availability of social robots, that is, robots devoted primarily to interact with human interlocutors. Examples of museum tour-guide robots (Nourbakhsh et al., 1999) and robots that interact with the elderly (Montemerlo et al., 2002) prove the advantages of social robots. However, they also pose the awareness of the need of natural and ecologic interactions. Many of these robots incorporate some rudimentary emotional behaviors. Robots with infant-like abilities of interaction were presented (e.g., Kismet by Breazeal, 2003) and used also to demonstrate the ability of people to understand and respond correctly to a robot’s display of emotions. Emotionally expressive graphical robot’s face encourages interactions with a robot (Bruce et al., 2002).

Therefore, there are several advantages that could derive from the use of thermal IR imaging for HMI. From the point of view of the computational physiology, there is the concrete possibility of monitoring, in a realistic environment, at a distance and unobtrusively, several physiological parameters and vital signs such as pulse rate, breathing rate, cutaneous vasomotor control, and indirect estimation of electro-dermal activity. This opens the way for remote monitoring of the physiological state of individuals without requiring their collaboration and without interfering with their usual activities, thus favoring the use of assistive robots. Another relevant possibility is to capitalize on thermal IR imaging to provide AA with the capability of adopting behavioral or communicative strategies contingent with the actual psychophysiological state of the human interface. This possibility, even though still not completely available, could be particularly effective for affective robots and automatic agents designed for improving and personalizing learning or treatment strategies on the basis of the measured user’s psychophysiological feedback.

Also, the technologically mediated interaction could be re-designed through the possibilities offered by thermal IR imaging, as it has been proved that collective emotions in cyberspace can be recorded and classified (Kappas, 2013). Participants communicating in real time via a computer exhibited expression and electro-dermal activations according to how well they got acquainted with each other in these interactions. They were physically separated, but online connected via text-based computer-mediated communication (Kappas et al., 2012). These processes emerge in real time and they apparently apply to e-communities of considerable size (Chmiel et al., 2011).

Of course, thermal IR imaging is not the first and unique attempt to endow the AA with the capability of understanding the affective and emotional state of the human interlocutor. This problem is well known to the robotic community (Pantic and Rothkrantz, 2003). Multimodal user-emotion detection systems for social robots have been presented. Alonso-Martín et al. (2013) recently proposed the robotics dialog system (RDS). This system uses two channels of information to detect emotional state: voice and face expression analysis. For emotion detection in facial expressions, the authors developed the gender and emotion facial analysis (GEFA). This system integrates two-party solutions: the first one recognizes the object in the field of view (SHORE – Sophisticated High-speed Object Recognition Engine) and the second one the facial expressions (CERT – Computer Expression Recognition Toolbox). The outcome of these components feed a decision rule to combine the information given by both of them to define the detected emotion.

Cid et al. (2014) presented Muecas, a multi-sensor humanoid robotic head for human–robot interaction. Muecas uses the mechanisms of perception and imitation of human expressions and emotions. These mechanisms allow direct interaction through different natural language modalities: speech, body language, and facial expressions. Muecas can be directly controlled by Facial Action Coding System (FACS), which is defined by the authors as “practically the standard for facial expression recognition and synthesis.”

**FIGURE 2:**
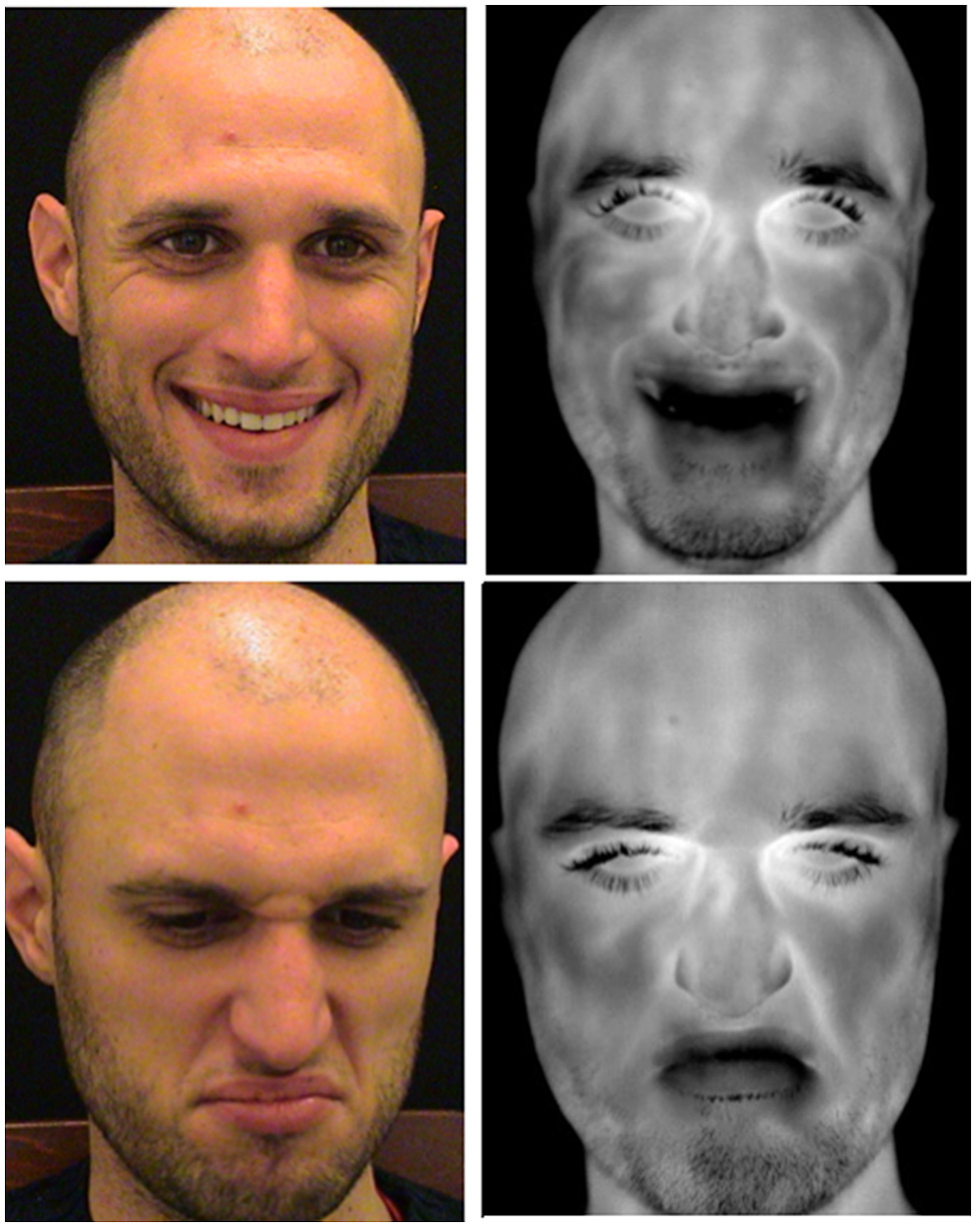
**Visible and thermal facial imprints of happiness (upper panel) and disgust (lower panel).** Thermal infrared (IR) imaging provides physiological response in addition to the behavioral ones measured through facial expression. Changes into the temperature distribution associated with the two different conditions could help in classifying affective states.

The use of behavioral responses, like speech, body language, and facial expressions, appears to be the most natural for classifying the human interlocutor affective state. However, the amount of information about the physiological state of the human interlocutor derived from his/her behavioral response is limited or absent at all. In this perspective, thermal IR imaging provides an extraordinary opportunity to add physiological information to behavioral responses for a better classification of affective states and emotional responses (**Figure 2**).

The above-mentioned studies, and the capability of thermal IR imaging of providing computational physiology data (Merla and Romani, 2007; Shastri et al., 2009; Merla, 2013), makes this technique a powerful tool for studying the psychophysiology of interpersonal relationships and intersubjectivity.

As the automatic recording and real-time processing of thermal IR imaging data for psychophysiology in realistic scenario is possible (Buddharaju et al., 2005; Dowdall et al., 2006; Merla et al., 2011), it seems that this technology, in combination or in addition with the other existing technologies, could potentially contribute to endow AA with the capability of monitoring the psychophysiological state of the human interlocutor. The technology and knowledge for achieving this result are available and already implemented in patent care and other applications (Merla, 2013).

Real-time processing of thermal IR imaging data and data classification for psychophysiological applications is possible as the computational demand is not larger than that required for 640 × 480 pixels visible-band imaging data (Buddharaju et al., 2005; Dowdall et al., 2006; Merla, 2014).

A major issue that needs to be addressed for a real use of thermal IR imaging in HMI is how specific method is for identifying specific emotional states at individual level. There are no specific studies available at the moment to answer such an important question, which remains matter of further research. A global limitation derives from the fact that cutaneous thermal activity is intimately linked to the autonomic activity. The question therefore becomes: “how specific and descriptive of each emotion are the autonomic responses?” A universally accepted answer is currently not available. Also no extensive studies are available about the fascinating possibility of merging together physiological information and automatic recognition of facial expressions for providing an atlas of the thermal signatures of emotions.

However, to date, no known attempts have been so far performed to integrate thermal IR imaging in any available system for robotic recognition of human affective state. Therefore, this opportunity remains a fascinating but still speculative possibility that needs to be validated with real-field studies.

## Conflict of Interest Statement

The author declares that the research was conducted in the absence of any commercial or financial relationships that could be construed as a potential conflict of interest.

## References

[B1] Alonso-Martín F. Malfaz M. Sequeira J. Gorostiza J. F. Salichs M. A. (2013). A multimodal emotion detection system during human–robot interaction. *Sensors* 13 15549–15581 10.3390/s131115549 24240598 PMC3871074

[B2] Breazeal C. (2003). Emotion and sociable humanoid robots. *Int. J. Hum. Comp. Stud.* 59 119–155 10.1016/S1071-5819(03)00018-1

[B3] Bruce A. Nourbakhsh I. Simmons R. (2002). “The role of expressiveness and attention in human–robot interaction,” in *Proceedings of the IEEE International Conference on Robotics and Automation, ICRA* Washington, DC

[B4] Buddharaju P. Dowdall J. Tsiamyrtzis P. Shastri D. Pavlidis I. Frank M. G. (2005). “Automatic Thermal Monitoring System (ATHEMOS) for Deception Detection,” in *Proceedings of the IEEE Computer Society Conference on Computer Vision and Pattern Recognition* Vol. 2 (Washington, DC: IEEE Computer Society) 1179

[B5] Butler E. A. Egloff B. Wilhelm F. H. Smith N. C. Erickson E. A. Gross J. J. (2003). The social consequences of expressive suppression. *Emotion* 3 48–67 10.1037/1528-3542.3.1.48 12899316

[B6] Chmiel A. Sienkiewicz J. Thelwall M. Paltoglou G. Buckley K. Kappas A. (2011). Collective emotions online and their influence on community life. *PLoS ONE* 6:e22207 10.1371/journal.pone.0022207 PMC3144870 21818302

[B7] Cid F. Moreno J. Bustos P. Núñez P. (2014). Muecas: a multi-sensor robotic head for affective human robot interaction and imitation. *Sensors* 14 7711–7737 10.3390/s140507711 24787636 PMC4063071

[B8] Clark H. H. Brennan S. E. (1991). “Grounding in communication,” in *Perspectives on Socially Shared Cognition,* eds Resnick L. B. Levine J. M. Teasley S. D. (Washington, DC: American Psychological Association) 127–149 10.1037/10096-006

[B9] Coli M. Fontanella L. Ippoliti L. Merla A. (2007). “Multiresolution KLE of psycho-physiological signals,” in *Proceedings of S.Co. 2007, Book of Short Papers* ISBN 978-886129-114-0 Venice, Padova 116–121

[B10] Di Giacinto A. Brunetti M. Sepede G. Ferretti A. Merla A. (2014). Thermal signature of fear conditioning in mild post traumatic stress disorder. *Neuroscience* 266 216–223 10.1016/j.neuroscience.2014.02.009 24561216

[B11] Dowdall J. B. Pavlidis I. T. Tsiamyrtzis P. (2006). “Coalitional tracking in facial infrared imaging and beyond,” in *Proceedings of the IEEE Computer Society Conference on Computer Vision and Pattern Recognition* Category number P2597

[B12] Ebisch S. J. Aureli T. Bafunno D. Cardone D. Romani G. L. Merla A. (2012). Mother and child in synchrony: thermal facial imprints of autonomic contagion. *Biol. Psychol.* 89 123–129 10.1016/j.biopsycho.2011.09.018 22001267

[B13] Engert V. Merla A. Grant J. A. Cardone D. Tusche A. Singer T. (2014). Exploring the use of thermal infrared imaging in human stress research. *PLoS ONE* 9:e90782 10.1371/journal.pone.0090782 PMC3968009 24675709

[B14] Forgas J. P. (1999). Feeling and speaking: mood effects on verbal communication strategies. *Personal. Soc. Psychol. Bull.* 25 850–863 10.1177/0146167299025007007

[B15] Frijda N. H. (2005). Emotion experience. *Cogn. Emot.* 19 473–497 10.1080/02699930441000346

[B16] Garbey M. Sun N. Merla A. Pavlidis I. T. (2007). Contact-free measurement of cardiac pulse based on the analysis of thermal imagery. *IEEE Trans. Biomed. Eng.* 54 1418–1426 10.1109/TBME.2007.891930 17694862

[B17] Hahn A. C. Whitehead R. D. Albrecht M. Lefevre C. E. Perret D. I. (2012). Hot or not? Thermal reactions to social contact. *Biol. Lett.* 8 864–867 10.1098/rsbl.2012.0338 22647931 PMC3440979

[B18] Hines E. A. Brown G. E. (1932). A standard stimulus for measuring vasomotor reactions: its application in the study of hypertension. *Proc. Staff Meet. Mayo Clin.* 7 332

[B19] Kappas A. (2013). Social regulation of emotion: messy layers. *Front. Psychol.* 4:51 10.3389/fpsyg.2013.00051 PMC3573210 23424049

[B20] Kappas A. Küster D. Theunis M. Tsankova E. (2012). Cyberemotions: an analysis of synchronous computer mediated communication and dyadic interaction. *Psychophysiology* 49 S49.

[B21] Khan M. M. Ward R. D. (2009). Classifying pretended and evoked facial expressions of positive and negative affective states using infrared measurement of skin temperature. *ACM Trans. Appl. Percept.* 6 1 10.1145/1462055.1462061

[B22] Kirby R. Forlizzi J. Simmons R. (2010). Affective social robots. *Rob. Auton. Syst.* 58 322–332 10.1016/j.robot.2009.09.015

[B23] Kirschbaum C. Pirke K. M. Hellhammer D. H. (1993). The ‘Trier Social Stress Test’ – a tool for investigating psychobiological stress responses in a laboratory setting. *Neuropsychobiology* 28 76–81 10.1159/000119004 8255414

[B24] Manini B. Cardone D. Ebisch S. J. Bafunno D. Aureli T. Merla A. (2013). Mom feels what her child feels: thermal signatures of vicarious autonomic response while watching children in a stressful situation. *Front. Hum. Neurosci.* 7:299 10.3389/fnhum.2013.00299 PMC3691510 23805091

[B25] Merla A. (2013). “Advances in thermal imaging for monitoring physiology and social interaction unobtrusively,” in *proceedings of the International Conference on Psychophysiology* Chieti–Pescara 50:S14

[B26] Merla A. (2014). “Revealing psychophysiology and emotions through thermal infrared imaging,” in *Proceedings of the International Conference on Physiological Computing Systems.* 368–377 10.5220/0004900803680377

[B27] Merla A. Cardone D. Di Carlo L. Di Donato L. Ragnoni A. Visconti A. (2011). “Noninvasive system for monitoring driver’s physical state,” in *Proceedings of the 11th AITA Advanced Infrared Technology and Applications* Abstract book, 21

[B28] Merla A. Di Donato L. Rossini P. M. Romani G. L. (2004). Emotion detection through functional infrared imaging: preliminary results. *Biomed. Tech.* 48 284–286

[B29] Merla A. Romani G. L. (2007). Thermal signatures of emotional arousal: a functional infrared imaging study. *Proc. Annu. Int. Conf. Proc. IEEE Eng. Med. Biol. Soc.* 2007 247–249 10.1109/IEMBS.2007.4352270 18001936

[B30] Mizukami K. Kobayashi N. Ishii T. Iwata H. (1990). First selective attachment begins in early infancy: a study using telethermography. *Infant Behav. Dev.* 13 257–271 10.1016/0163-6383(90)90034-6

[B31] Montemerlo M. Pineau J. Roy N. Thrun S. Verma V. (2002). “Experiences with a mobile robotic guide for the elderly,” in *Proceedings of the National Conference of Artificial Intelligence (AAAI 02)* Edmonton, AB

[B32] Murthy R. Pavlidis I. T. (2006). Noncontact measurement of breathing function. *IEEE Eng. Med. Biol. Mag.* 25 57–67 10.1109/MEMB.2006.1636352 16764432

[B33] Nhan A. R. Chau T. (2010). Classifying affective states using thermal infrared imaging of the human face. *IEEE Trans. Biomed. Eng.* 57 979–987 10.1109/TBME.2009.2035926 19923040

[B34] Nourbakhsh I. Bobenage J. Grange S. Lutz R. Meyer R. Soto A. (1999). An affective mobile robot educator with a full-time job. *Artif. Intell.* 114 95–124 10.1016/S0004-3702(99)00027-2

[B35] Pantic M. Rothkrantz L. J. M. (2003). Toward an affect-sensitive multimodal human-computer interaction. *Proc. IEEE* 91 1370–1390 10.1109/JPROC.2003.817122

[B36] Parkinson A. (1996). Emotions are social. *Br. Psychol. Soc.* 87 663–683 10.1111/j.2044-8295.1996.tb02615.x 8962482

[B37] Pavlidis I. T. Dowdall J. Sun N. Puri C. Fei J. Garbey M. (2007). Interacting with human physiology. *Comput. Vis. Image Underst.* 108 150–170 10.1016/j.cviu.2006.11.018

[B38] Pavlidis I. Eberhardt N. L. Levine J. A. (2002). Human behavior: seeing through the face of deception. *Nature* 415 35 10.1038/415035a 11780104

[B39] Pavlidis I. T. Tsiamyrtzis P. Shastri D. Wesley A. Zhou Y. Lindner P. (2012). Fast by nature – how stress patterns define human experience and performance in dexterous tasks. *Sci. Rep.* 2 1–9 10.1038/srep00305 PMC3294268 22396852

[B40] Pollina D. A. Dollins B. A. Senter S. M. Brown T. E. Pavlidis I. T. Levine J. A. (2006). Facial skin surface temperature changes during a “concealed information” test. *Ann. Biomed. Eng.* 34 1182–1189 10.1007/s10439-006-9143-3 16786391

[B41] Reeves B. Nass C. (1996). *The Media Equation.* Cambridge: CSLI Publications

[B42] Ring E. F. J. Ammer K. (2012). Infrared thermal imaging in medicine. *Physiol. Meas.* 33 R33–R46 10.1088/0967-3334/33/3/R33 22370242

[B43] Shastri A. Merla A. Tsiamyrtzis P. Pavlidis I. T. (2009). Imaging facial signs of neurophysiological responses. *IEEE Trans. Biomed. Eng.* 56 477–484 10.1109/TBME.2008.2003265 19272941

[B44] Tsiamyrtzis P. Dowdall J. Shastri D. Pavlidis I. T. Frank M. G. Ekman P. (2006). Imaging facial physiology for the detection of deceit. *Int. J. Comput. Vis.* 71 197–214 10.1007/s11263-006-6106-y

[B45] Wild B. Erb M. Bartels M. (2001). Are emotions contagious? Evoked emotions while viewing emotionally expressive faces: quality, quantity, time course and gender differences. *Psychiatry Res.* 102 109–124 10.1016/S0165-1781(01)00225-6 11408051

[B46] Zhou Y. Tsiamyrtzis V. Pavlidis I. T. (2009). Tissue tracking in thermo-physiological imagery through spatio-temporal smoothing. *Med. Image Comput. Comput. Assist. Interv.* 12 1092–1099 20426220 10.1007/978-3-642-04271-3_132

